# Lactate dehydrogenase-A inhibition induces human glioblastoma multiforme stem cell differentiation and death

**DOI:** 10.1038/srep15556

**Published:** 2015-10-23

**Authors:** Simona Daniele, Chiara Giacomelli, Elisa Zappelli, Carlotta Granchi, Maria Letizia Trincavelli, Filippo Minutolo, Claudia Martini

**Affiliations:** 1 Department of Pharmacy, University of Pisa, 56126 Pisa, Italy

## Abstract

Therapies that target the signal transduction and metabolic pathways of cancer stem cells (CSCs) are innovative strategies to effectively reduce the recurrence and significantly improve the outcome of glioblastoma multiforme (GBM). CSCs exhibit an increased rate of glycolysis, thus rendering them intrinsically more sensitive to prospective therapeutic strategies based on the inhibition of the glycolytic pathway. The enzyme lactate dehydrogenase-A (LDH-A), which catalyses the interconversion of pyruvate and lactate, is up-regulated in human cancers, including GBM. Although several papers have explored the benefits of targeting cancer metabolism in GBM, the effects of direct LDH-A inhibition in glial tumours have not yet been investigated, particularly in the stem cell subpopulation. Here, two representative LDH-A inhibitors (**NHI-1** and **NHI-2**) were studied in GBM-derived CSCs and compared to differentiated tumour cells. LDH-A inhibition was particularly effective in CSCs isolated from different GBM cell lines, where the two compounds blocked CSC formation and elicited long-lasting effects by triggering both apoptosis and cellular differentiation. These data demonstrate that GBM, particularly the stem cell subpopulation, is sensitive to glycolytic inhibition and shed light on the therapeutic potential of LDH-A inhibitors in this tumour type.

Glioblastoma multiforme (GBM), a WHO (World Health Organization) grade IV astrocytoma, is the most common and aggressive primary brain tumour in adults, with a median survival of less than 12 months due to its radio- and chemoresistance^1^,^2^. The persistence of residual disease and recurrence can be partially explained by the failure to eradicate a subset of cells within the tumour, called cancer stem cells (CSCs). Indeed, CSCs identified in several human malignancies are intrinsically more resistant to chemotherapeutic agents and radiation than the bulk of the tumour cells^3^,^4^.

Like other cancers, GBM requires a continuous source of energy and molecular resources for new cell production. An excessive conversion of glucose to lactate, a higher rate of glycolysis and a reduction of pyruvate oxidation are the hallmarks of several cancers, even in the presence of ample oxygen levels. This metabolic change is recognized as the Warburg effect^5^,^6^. For these reasons, interventions targeting the glycolysis-induced metabolic reprogramming likely constitute a promising approach for the treatment of GBM^7^,^8^. Moreover, recent findings have demonstrated that glioma stem cells (GSCs) exhibit an increased rate of glycolysis and low mitochondrial respiratory activity and prefer a hypoxic microenvironment to maintain their stemness^9^, thus rendering them intrinsically more sensitive to prospective therapeutic strategies based on the inhibition of the glycolytic pathway^10^,^11^,^12^. In this respect, dichloroacetate (DCA), an inhibitor of pyruvate dehydrogenase kinase, has been proven to shift the pyruvate metabolism in rat GSCs^13^.

Recently, interventions targeting lactate metabolism are emerging as a promising approach for cancer therapy^14^,^15^. Lactate dehydrogenase (LDH) mediates the bidirectional conversion of pyruvate and lactate and constitutes a major checkpoint for the switch from oxidative phosphorylation (OXPHOS) to glycolysis. LDH is a tetrameric enzyme composed of two different subunits LDH-A (LDH-M, muscle) and LDH-B (LDH-H, heart), which can differentially assemble into five different isoforms. While LDH-B_4_ (LDH1) is ubiquitously expressed, LDH-A_4_ (LDH5) is the predominant isoform found in skeletal muscle and other highly glycolytic tissues, and has a higher affinity for pyruvate, as well as a higher *V*_max_ for pyruvate reduction than LDH-B_4_.

Elevated levels of LDH-A are a hallmark of many tumours, including GBM, and correlate to a poor prognosis in several human malignancies^16^,^17^,^18^, to the point that altered serum and plasma LDH-A levels have been proposed as a possible biomarker of different types of cancer^19^,^20^.

LDH-A is thought to be a major molecular mediator of the Warburg effect and to play a critical role in sustaining cancer’s glycolytic phenotype. Notably, LDH-A suppression has been reported to reduce tumour progression^21^,^22^ and the *in vivo* growth of transplanted breast tumours^21^,^22^,^23^. Further, FX11, a small-molecule inhibitor of LDH-A, impairs the growth of human pancreatic cancer and lymphoma xenografts^22^.

Although several papers have explored the benefits of targeting cancer metabolism in GBM^24^, the effects of direct LDH-A inhibition have not yet been explored, particularly in the stem cell population. In this respect, Xie and collaborators recently demonstrated that down-regulation of LDH-A activity reduced the stem cell population of lung carcinoma cells^25^, thus supporting a mechanistic rationale by which LDH-A inhibition could be a viable therapeutic target for CSCs.

A previous work described a series of *N*-hydroxyindole–based compounds^26^,^27^ that were demonstrated to act as selective LDH-A inhibitors. These agents impair the proliferation of cancer cell lines and primary cancer cultures with micromolar IC_50_ values^26^,^28^,^29^,^30^,^31^.

In this report, two representative LDH-A inhibitors (NHI-1 and NHI-2) were used as tools to investigate the effects of LDH inhibition in different GBM cell lines and in GSCs. These LDH-A inhibitors decreased GBM cell proliferation, triggered cellular apoptosis and blocked the cell cycle. Most importantly, LDH-A inhibition was more effective in GSCs, where the two compounds elicited long-lasting effects by triggering both apoptosis and cell differentiation. Globally, our findings demonstrated that GBM, particularly the GSC subpopulation, is sensitive to glycolytic inhibition, and shed light on the therapeutic potential of LDH-A inhibitors in this tumour, which is characterised by a poor prognosis.

## Results

### Effects of LDH-A inhibition on U87MG cell viability

**Figure 1:**
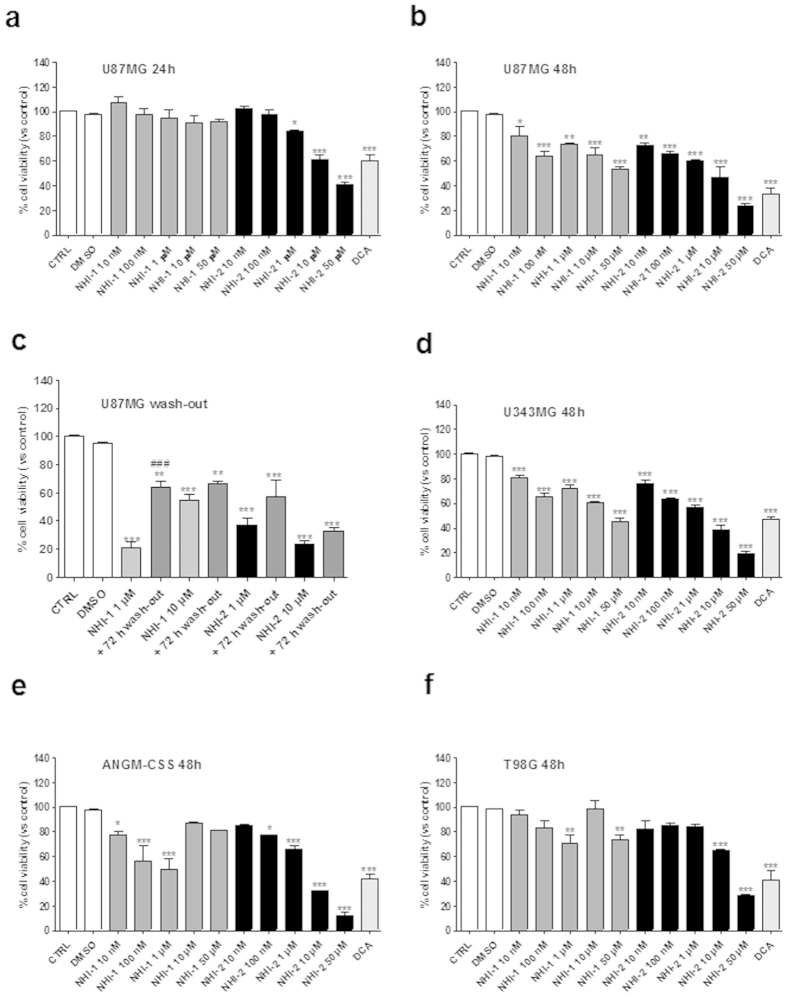
Effects of LDH-A inhibition on GBM cell viability. U87MG cells were treated in complete medium with the indicated concentrations of NHI-1 or NHI-2 or DCA (100 μM) for 24 h (**a**), or 48 h (**b**). (**c**) U87MG cells were treated with NHI-1 or NHI-2 (1 μM or 10 μM) for 72 h, and subsequently the cells were washed-out for additional 72 h in drug-free medium. U343MG (**d**), ANGM-CSS (**e**) or T98G cells (**f**) were treated in complete medium with the indicated concentrations of NHI-1 or NHI-2 or DCA (100 μM) for 48 h. At the end of the treatments, cell viability was measured using CellTrace dye labelling, as described in the Methods section. The data were expressed as the percent change with respect to untreated cells (control), which was set to 100%, and they are the mean values ± SEM of three independent experiments, each performed in triplicate. The significance of the differences was determined by one-way ANOVA and Bonferroni’s post hoc test: *P ≤ 0.05, **P ≤ 0.01, ***P ≤ 0.001 vs. the control; ###P ≤ 0.001 vs. the cells treated for 72 h.

The cellular and molecular mechanisms related to LDH-inhibition were first investigated in GBM cells, and subsequently in GBM-derived GCSs. To examine the effects of representative NHI-based LDH-A inhibitors NHI-1 and NHI-2 on GBM cell viability, U87MG cells were treated with different concentrations of LDH-A inhibitors (10 nM − 50 μM) for 24 or 48 h. Both compounds decreased U87MG viable cells in a time-dependent manner (Fig. 1a,b), with a maximal effect comparable to that elicited by DCA (100 μM), a compound capable of shifting metabolism from glycolysis to glucose oxidation, via an inhibition of pyruvate dehydrogenase kinase^32^. In addition, NHI-2 showed a dose-dependent inhibitory effect on U87MG cells at each time of treatment, yielding IC_50_ values of 4.66 ± 0.51 μM and 842 ± 78 nM after 24 h and 48 h of incubation, respectively (Supplementary Figure 1). To assess whether U87MG cells could resume proliferation, cells were treated for 72 h with the compounds (1 μM or 10 μM). At the end of the treatment periods, the culture medium was replaced with fresh medium not containing the drug. As depicted in Fig. 1c, U87MG partially recovered their growth after 72 h of washout, although the percentage of proliferation remained significantly reduced with respect to control cells. These results suggest that compounds are not able to block completely the proliferation of U87MG cells.

In order to extend our observations on the therapeutically potential of LDH-A inhibitors in GBM, viability experiments were repeated in different GBM cell lines, displaying either a wild-type p53 (i.e., U343MG, ANGM-CSS cells^33^,^34^) or a mutated p53 (T98G cells^35^). As depicted in Fig. 1d,e, both compounds NHI-1 and NHI-2 lead to a significant inhibition of the viability of U343MG and ANGM-CSS cells, similarly to what observed in U87MG cells. Notably, the percentage of inhibition elicited by these LDH-A inhibitors on T98G cell viabilitys were slightly lower than those obtained in other GBM cell lines (Fig. 1f), thus suggesting that LDH-A-mediated effects involved, at least partially, the p53 pathway.

### Effects of LDH-A inhibition on the induction of U87MG apoptosis and cell cycle block

**Figure 2:**
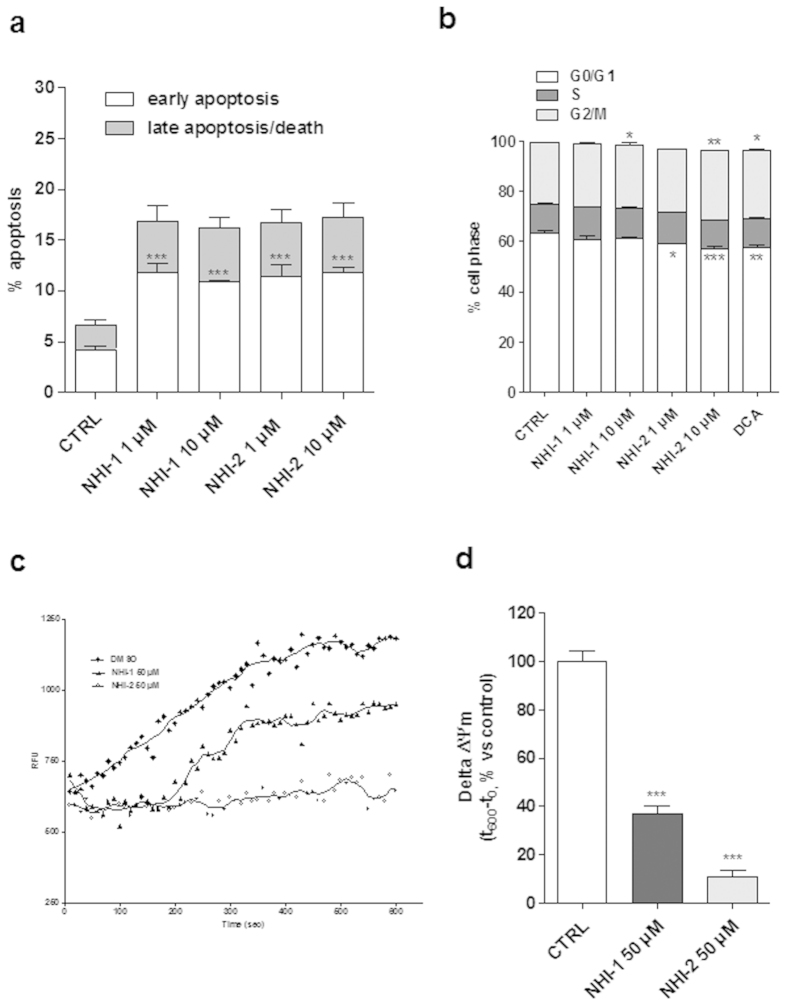
Effects of LDH-A inhibition on U87MG cell apoptosis, cell cycle progression and Δψm. (**a**) U87MG cells were treated for 48 h with DMSO (control), or NHI-1 or NHI-2 at 1 μM or 10 μM. At the end of the treatment periods, the cells were collected and the level of phosphatidylserine externalisation was evaluated using the Annexin V-staining protocol. The data were expressed as the percentage of apoptotic cells versus the total number of cells, and are the mean ± SEM of three different experiments. (**b**) U87MG cells were treated for 72 h with DMSO (control), or NHI-1 or NHI-2 at 1 μM or 10 μM, or DCA at 100 μM and the cell cycle was analysed. The data were expressed as percentage of cell in the different phases (G0/G1, G2/M or S) versus total cell number, and they are the mean values ± SEM of three different experiments. (**c**,**d**) U87MG were treated for 48 h with DMSO (control) or 50 μM NHI-1 or NHI-2. At the end of treatments, the mitochondria were isolated and the Δψm (5 μg of proteins) was evaluated using JC-1 protocol (**c**) Representative graph of the mitochondrial potential analysis using the JC-1 protocol. (**d**) The data were expressed as the variation of JC-1 uptake into mitochondria, which was calculated as the difference between the RFU at the beginning and those read after 10 min, and represent the means ± SEM of three different experiments, each performed in duplicate. The significance of the differences was determined by one-way ANOVA and Bonferroni’s post hoc test: *P ≤ 0.05, **P ≤ 0.01, ***P ≤ 0.001 vs. the control.

We then investigated whether the reduction in the number of viable cells elicited by the LDH-A inhibitors could be associated with apoptosis. Treating of the cells with NHI-1 or NHI-2 (1 μM or 10 μM) for 48 h caused a significant amount of phosphatidylserine externalisation, in the absence of 7-aminoactinomycin D (7-AAD) binding to DNA (Fig. 2a and Supplementary Figure 2A), demonstrating that the compounds mainly induced early apoptosis of U87MG cells.

Next, the effects of the LDH-A inhibitors on cell cycle were assessed. As depicted in Fig. 2b and Supplementary Figure 2B, both compounds induced cell cycle disturbance. In particular, NHI-2 arrested cell-cycle in the G2/M phase at both tested concentrations (1 μM and 10 μM); in contrast, NHI-1, only at the higher concentration, caused a slight increase of the G2/M subpopulation. Similarly, DCA (100 μM) induced cell cycle arrest in G2/M, and this effect was comparable to that obtained with the highest concentration of compound NHI-2 (Fig. 2b and Supplementary Figure 2B).

### Effects of LDH-A inhibition on mitochondrial membrane potential (Δψm) in U87MG cells

Because a reduction in LDH-A activity has been related to a stimulation of mitochondrial respiration and a decrease of membrane potential^23^, the effects of LDH-A inhibitors on mitochondrial depolarization (Δψm) were assessed. U87MG were treated with a high concentration (50 μM) of NHI-1 or NHI-2 for 48 h and then Δψm was measured in isolated mitochondria. The results showed that both NHI-1 and NHI-2 markedly reduced JC-1 accumulation in mitochondria isolated from U87MG cells (Fig. 2c,d), confirming collapse of Δψm after LDH-A inhibition.

### Effects of LDH-A inhibition on GSC viability

Recent evidence has demonstrated that CSCs show higher expression levels and activity of key enzymes of anaerobic glucose metabolism^10^, including LDH-A^36^, thus suggesting that LDH-A may be a promising pharmacological target to counteract the chemoresistance of these cells. Therefore, the effects of LDH-A inhibitors on GSCs isolated from GBM cells were evaluated. The formation of neurospheres in GBM cell cultures *in vitro* was induced by a specific neural stem-cell (NSC) medium^37^. Cell spheres have been described to be richer in tumour stem cells than the cells directly attached to the culture flask (called monolayers^38^; see Supplementary Figure 3A). Accordingly, the spheres obtained using U87MG cells included significantly more CD133/Nestin^+^ cells and a smaller percentage of GFAP^+^ cells compared with the pool of whole U87MG cells, as demonstrated using real-time PCR and Western blotting analysis (Supplementary Figure 3B,C,D). Similar results were confirmed in U343MG, ANGM-CSS and T98G cells (Supplementary Figure 4).

**Figure 3:**
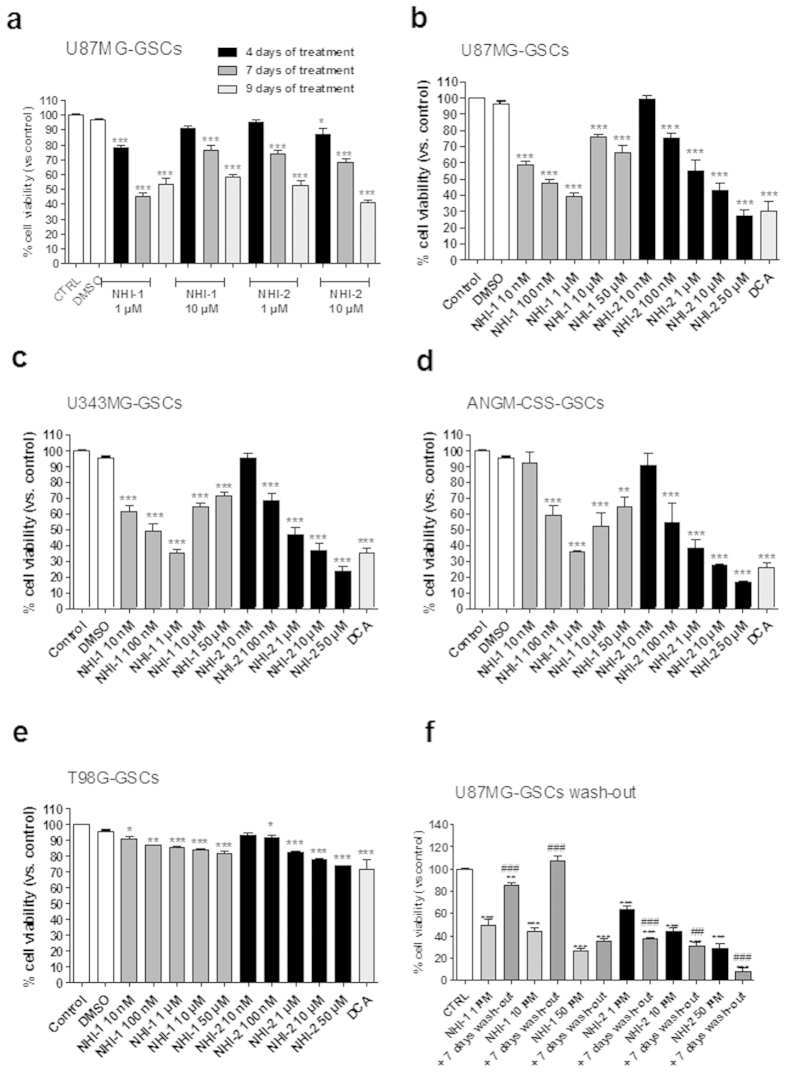
Effect of LDH-A inhibition on GSC viability. GSCs isolated from U87MG (**a**), U343MG (**b**), ANGM-CSS (**c**) or T98G (**d**) were incubated with different concentrations of NHI-1 or NHI-2 or DCA (100 μM) for the indicated days. (**e**) U87MG-GSCs were treated with NHI-1 or NHI-2 (1 μM, 10 μM) for 7 days, and subsequently the medium was replaced with drug-free fresh medium for another 7 days. At the end of treatments, cell proliferation was measured using CellTrace dye labelling, as described in the Methods section. The data were expressed as the percent change with respect to the untreated cells (control), which was set to 100%, and they are the mean values ± SEM of three independent experiments, each performed in duplicate. The significance of the differences was determined by one-way ANOVA and Bonferroni’s post hoc test: *P ≤ 0.05, **P ≤ 0.01, ***P ≤ 0.001 vs. the control; ##P ≤ 0.01, ###P ≤ 0.001 vs. the cells treated for 72 h.

As depicted in Fig. 3a, the NHI-1 and NHI-2 compounds induced a time-dependent inhibition of U87MG-GSC viability at both tested concentrations, with a maximal effect comparable to that elicited by DCA (100 μM)^39^. The effects of compound NHI-2 appeared to be concentration dependent, with an IC_50_ value of 347 ± 39 nM after 7 days of cell incubation (Fig. 3b and Supplementary Figure 5). Similar results were obtained in GSC isolated from U343MG and ANGM-CSS cells (Fig. 3c,d). In contrast, NHI-1 and NHI-2 only slightly inhibited the viability of the GSCs isolated from T98G cells (Fig. 3e), thus suggesting that the p53 pathway may also have a notable role in LDH-A inhibition-mediated effects in the cancer stem cell subpopulation.

Next, we assessed whether the NHI-2 and/or NHI-1-treated cells could resume their growth after drug removal. After the U87MG-GSC challenging with compound NHI-2 (1 μM, 10 μM or 50 μM), and a wash-out period of 7 days, the percentages of viable cells did not significantly increase, suggesting their overall inability to recover normal growth (Fig. 3f). Compound NHI-1 showed long-lasting effects only when used at 50 μM (Fig. 3f). A comparison of these data with those obtained in wash-out experiments in U87MG cells suggests the ability of LDH-A inhibitors to preferentially produce long-lasting effects in the GSC subpopulation.

### Effects of LDH-A inhibition on GBM cancer sphere formation

**Figure 4:**
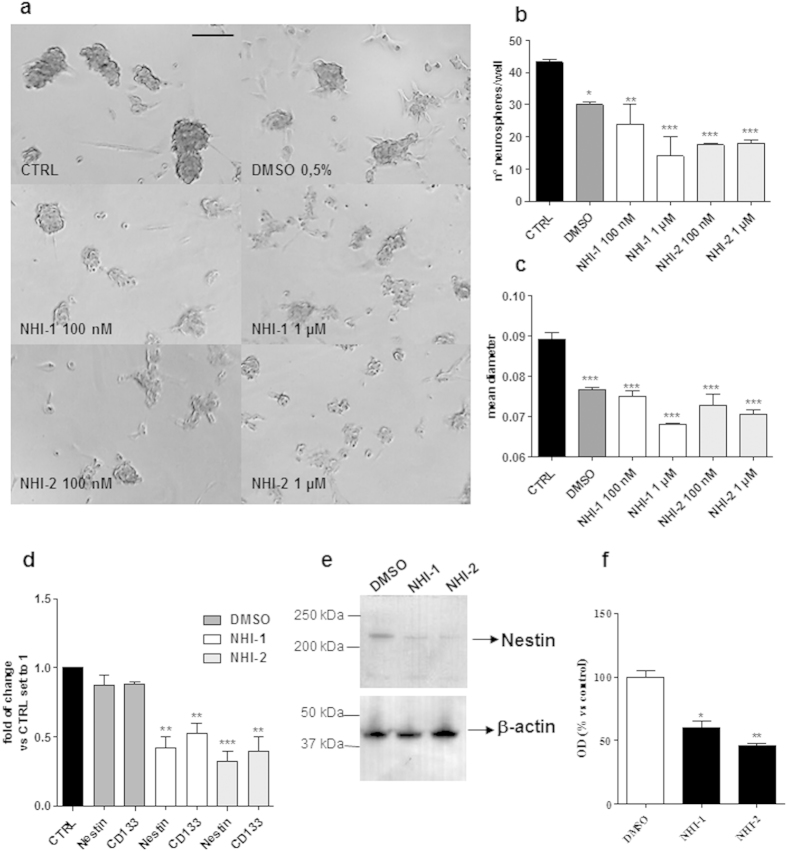
Effect of LDH-A inhibition on the formation of GSCs. U87MG were incubated with DMSO or the NHI-1 and NHI-2 compounds (100 nM−1 μM) in a defined serum-free NSC medium for 9 days. (**a**) Representative pictures of the cells after 9 days of incubation were shown. The number of the newly formed spheres (**b**) and the mean diameter (**c**) were scored using the ImageJ program. The data represent the means ± SEM of three pictures from two independent experiments. The significance of the differences was determined by one-way ANOVA and Bonferroni’s post hoc test: *P ≤ 0.05, **P ≤ 0.01, ***P ≤ 0.001 vs. the control or DMSO. (**d**) The cells were treated as in (**a**) (NHI-1 and NHI-2: 100 nM). Real Time RT-PCR analysis of the stem cell markers (CD133 and Nestin) was performed. (**e**,**f**) The cells were treated as in (**a**) (NHI-1 and NHI-2: 100 nM), and the obtained cell lysates were subjected to Western blotting. (**e**) Representative Western blots. The full-length blots are reported in the Supplementary Information section titled “Full-length blots of the cropped images shown in the main Figures”. (**f**) The bar graph shows the results of the quantitative analysis of the Western blots, which was performed using the ImageJ program. The data were expressed as the percent change in the optical density of the immunoreactive bands relative to that of the control, which was set to 100%, and are the mean values ± SEM of three different experiments. The significance of the differences was determined by one-way ANOVA and Bonferroni’s post hoc test: *P < 0.05, **P < 0.01 vs. control.

The ability of LDH-A inhibitors to affect the formation of GSCs was examined. To this purpose, adherent U87MG cells were switched to a defined serum-free NSC medium, and the cells were allowed to growth for 9 days in the presence or absence of the LDH-A inhibitors (Fig. 4a–c). NHI-1 and NHI-2 were able to decrease the diameter of the newly formed spheres, suggesting their ability to inhibit the proliferation of glioblastoma stem cells (Fig. 4c). Moreover, the compounds caused a reduction in the sphere number, indicating their capacity to also alter the stem cell-generating ability of the GBM cells (Fig. 4b). However, to more clearly demonstrate the effects on GSC formation, stem cell markers (CD133 and Nestin) were evaluated after the treatment with NHI-1 or NHI-2 at 100 nM, a concentration that is ten times lower than the IC_50_ values in the viability assay (Fig. 4d–f). As reported, both compounds were able to significantly decrease mRNA and protein expression of staminal markers, thus highlighting their ability to modulate not only the maintenance of cancer stem profile, but also the de-differentiation process.

### Effects of LDH-A inhibition on morphology and differentiation of GSCs

**Figure 5:**
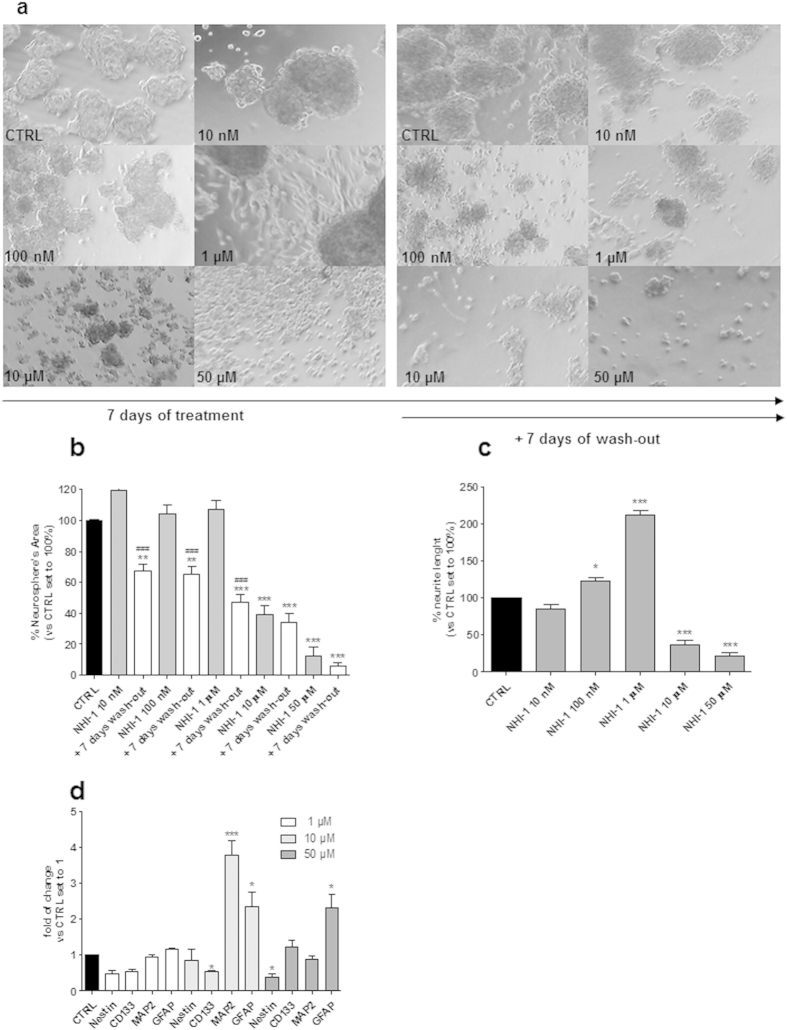
Effect of NHI-1 on the sphere-derived cell morphology. The GSCs were treated with complete NSC medium containing DMSO (control) or the indicated concentrations of NHI-1 for 7 days. As indicated, the drug-containing media were replaced with fresh drug-free NSC medium at the end of treatments, and the cells were cultured for additional 7 days. (**a**) Representative cell micrographs after 7 days of treatment and after 7 days of drug wash-out were shown. (**b**) The area of the culture plates occupied by the spheres and (**c**) the length of cellular processes were scored after 7 days of treatment and after 7 days of drug wash-out. The counts represent the mean values ± SEM of three independent experiments. The significance of differences was determined by one-way ANOVA and Bonferroni’s post hoc test: **P ≤ 0.01, ***P ≤ 0.001 vs. the control; ###P ≤ 0.001 vs. the GSCs treated for 7 days. (**d**) The GSCs were treated with complete NSC medium containing DMSO (control) or the indicated concentrations of NHI-1 for 7 days. At the end of treatments, the total RNA was extracted, and the relative mRNA levels of the stem cell marker CD133, the neuronal marker MAP2, and the astrocyte marker GFAP were quantified by real time RT-PCR. The data were expressed as the fold change compared to the levels of the control, which were set to 1, and are the mean values ± SEM of three different experiments. The significance of the differences was determined by one-way ANOVA and Bonferroni’s post hoc test: *P ≤ 0.05, ***P ≤ 0.001 vs. the control.

**Figure 6:**
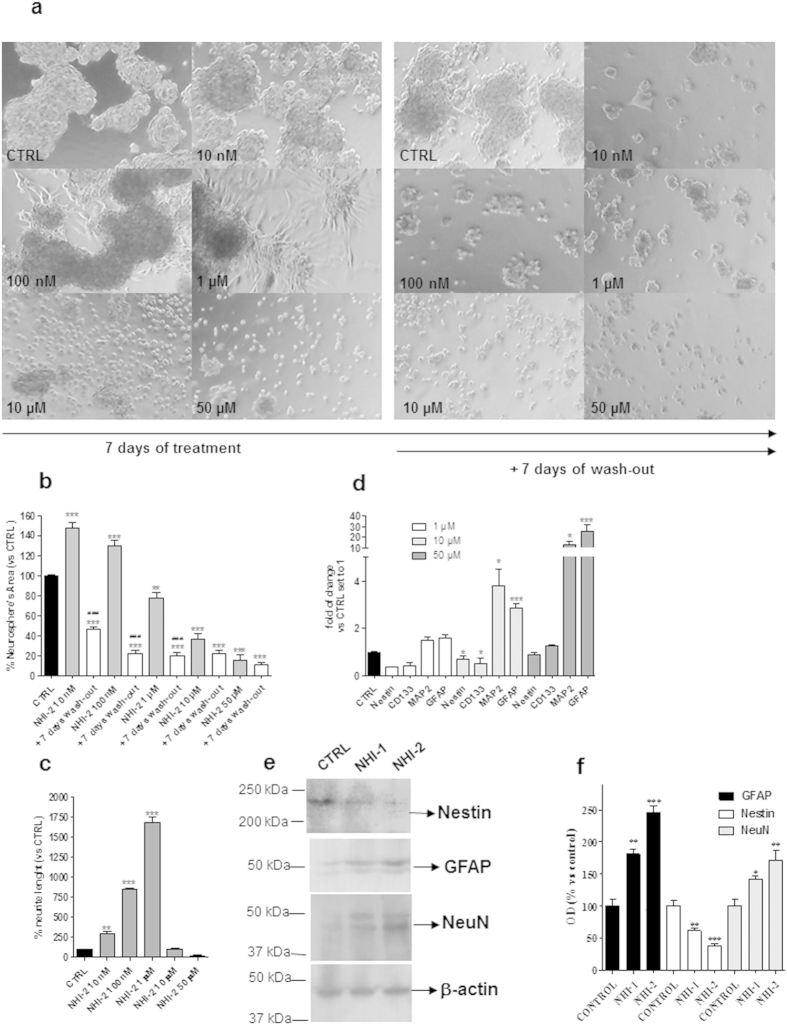
Effect of NHI-2 on the sphere-derived cell morphology. The GSCs were treated with complete NSC medium containing DMSO (control) or the indicated concentrations of NHI-2 for 7 days. The drug-containing media were replaced with fresh drug-free NSC medium at the end of the treatments, and cells were cultured for an additional 7 days. (**a**) Representative cell micrographs. (**b**) The area occupied by the spheres and (**c**) the length of cellular processes were scored after 7 days of treatment and after 7 days of drug wash-out. The counts represent the mean values ± SEM of three independent experiments. The significance of differences was determined by one-way ANOVA and Bonferroni’s post hoc test: **P ≤ 0.01, ***P ≤ 0.001 vs. the control; ###P ≤ 0.001 vs. the GSCs treated for 7 days without wash out. (**d**) The GSCs were treated with complete NSC medium containing DMSO (control) or the indicated concentrations of NHI-2 for 7 days. At the end of treatments, the relative mRNA levels of CD133, MAP2 and GFAP were quantified by real time RT-PCR. The data were expressed as the fold change vs. the levels of the control, which were set to 1, and are the mean values ± SEM of three different experiments. *P ≤ 0.05, ***P ≤ 0.001 vs. the control. (**e**,**f**) The GSCs were treated for 7 days with complete NSC medium containing DMSO (control) or NHI-1 or NHI-2 (10 μM). At the end of treatments, the obtained cell lysates were subjected to Western blotting. (**e**) Representative Western blots. The full-length blots of the cropped images shown in the main Figures are included in the Supplemental Information. (**f**) The bar graph shows the results of the quantitative analysis of the Western blots, which was performed using the ImageJ program. The data were expressed as the percent change in the optical density of the immunoreactive band relative to that of the control, which was set to 100%, and are the mean values ± SEM of three different experiments. **P ≤ 0.01, ***P ≤ 0.001 vs. the control.

The effects of NHI-1 and NHI-2 on GSC morphology were evaluated quantifying the area occupied by the cells in culture plates, as well as the outgrowth of cellular processes. Representative photographs of control and treated cells are shown in Fig. 5a and 6a. Both NHI-1 and NHI-2 (10 μM or 50 μM) reduced the area occupied by the floating spheres (Fig. 5a,b and 6a,b); these effects were notable even after cell wash-out. Of note, when GSCs were incubated with lower concentrations of the LDH-A inhibitors (i.e., 100 nM and 1 μM), a prominent outgrowth of cellular processes was noticed (Fig. 5a–c and 6a–c), demonstrating that inhibition of LDH-A could also induce GSC differentiation. To investigate the mechanisms through which LDH-A inhibitors affected GSC morphology, we then assessed the levels of stem and differentiation markers upon stimulation with the two compounds. In GSCs treated with NHI-1 and NHI-2 a significant decrease in the stemness markers, CD133 and Nestin, was noticed (Fig. 5d and 6d), accompanied by a significant increase of MAP2 and GFAP content. These data demonstrate that the LDH inhibition was able to promote GSC differentiation toward a neuronal and a glial phenotype. When NHI-1 and NHI-2 were used at high concentration (10 μM and 50 μM), the effects on neurite elongations were remarkably reduced. Surprisingly, despite no significant cellular processes had been noticed in the morphological analysis, a significant decrease in the stemness marker nestin or a marked increase in the MAP2 and GFAP content (Fig. 5d and 6d) was evidenced. These data were confirmed also in different GBM cell lines (Supplementary Figure 6). Moreover, western blotting analysis confirmed that both compounds at 10 μM induced a significant decrease in the stemness marker Nestin and an increase in glial and neuronal markers (GFAP and NeuN; Fig. 6e,f).

Taken together, these results highlighted the essential role of the LDH-A on the cancer stem cell balance between differentiation and death processes. NHI-1 and NHI-2 triggered the differentiation of GSCs and, notably, at higher concentrations, they also produced a simultaneous marked decrease of GSCs proliferation, thus hiding the morphological sign of differentiation.

### Effects of LDH-A inhibition on the induction of GSC apoptosis and cell cycle block

**Figure 7:**
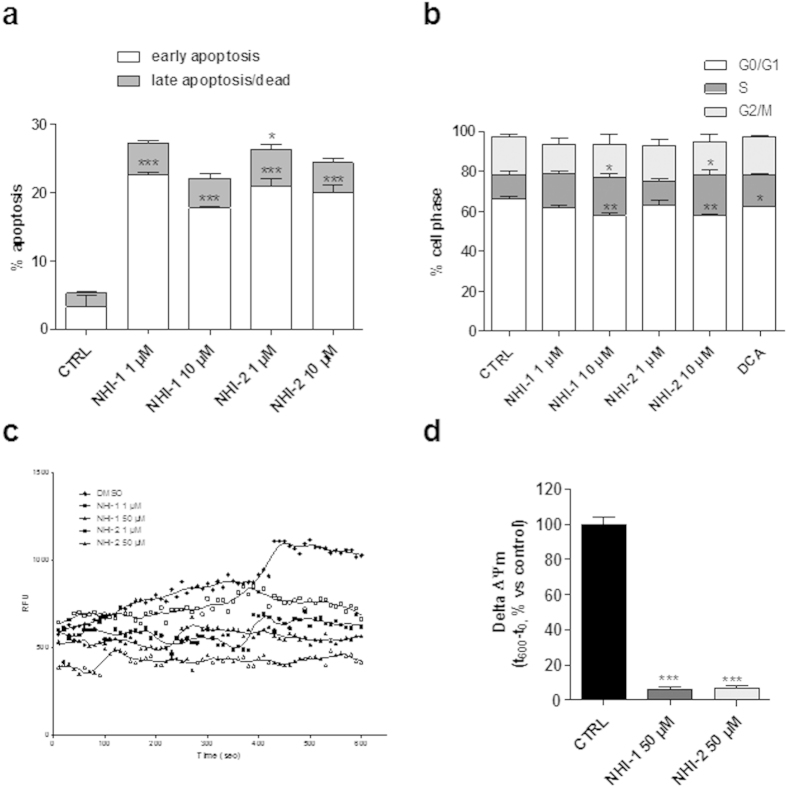
Effect of LDH-A inhibition on the induction of GSC apoptosis, cell cycle block, and Δψm. (**a**) GSCs were treated for 7 days with DMSO (control), or NHI-1 or NHI-2 (1 μM−10 μM). At the end of the treatments, the cells were collected and the level of phosphatidylserine externalisation was evaluated using the Annexin V-staining protocol. The data were expressed as the percentage of apoptotic cells versus the total number of cells, and represent the mean ± SEM of three different experiments. (**b**) GSCs were treated for 7 days with DMSO (control), or NHI-1 or NHI-2 (1 μM−10 μM), or DCA (100 μM) and the cell cycle was analysed. The data were expressed as percentage of cell in the different phases (G0/G1, G2/M or S) versus total cell number, and they are the mean values ± SEM of three different experiments. (**c**) GSCs were treated with DMSO or NHI-1 or NHI-2 (1 μM−50 μM) for 7 days. At the end of treatments, mitochondria were isolated and the Δψm (for 5 μg of proteins) was evaluated using JC-1 protocol. (**c**) Representative graph of mitochondria potential evaluation using JC-1 protocol. (**d**) The data were expressed as the variation of JC1 uptake into mitochondria, calculated as the difference between RFU at the beginning and those read after 10 min, and represent the mean ± SEM of three different experiments, each performed in duplicate. The significance of the differences is determined with a one-way ANOVA with Bonferroni’s post hoc test: *P ≤ 0.05, **P ≤ 0.01, ***P ≤ 0.001 vs. the respective control.

We then investigated if the reduction of GSC proliferation elicited by the two compounds could be associated to apoptosis. Neither NHI-1 nor NHI-2 induced GSC apoptosis after 4 days of treatment (data not shown). On the contrary, treating GSC with the LDH-A inhibitors (at 1 μM or 10 μM) for 7 days caused a significant phosphatidylserine externalization in the absence of 7-amino actinomycin binding to DNA, thus denoting the induction of early apoptosis (Fig. 7a and Supplementary Figure 7A). The comparison between morphological analysis and RT-PCR data indicated that the compounds triggered both apoptosis and differentiation of GSCs.

Cell cycle analysis demonstrated that challenging GSCs for 7 days with NHI-1 or NHI-2, tested at 10 μM, induced cell cycle arrest in G2/M phase decreasing significantly the G0/G1 subpopulation (Fig. 7b and Supplementary Figure 7B). Similarly, DCA (100 μM) significantly reduced the percentage of GSCs in G2/M phases (Fig. 7b and Supplementary Figure 7B).

### Effects of LDH-A inhibition on mitochondrial membrane potential (Δψm) in GSCs

The effects of LDH-A inhibitors on mitochondrial depolarization were assessed also in GSCs. GSCs were then treated with NHI-1 or NHI-2 for 7 days, and then Δψm was measured in isolated mitochondria. The results showed that both NHI-1 and NHI-2 markedly reduced JC-1 accumulation in mitochondria isolated from GSCs (Fig. 7c,d), confirming a collapse of Δψm after LDH-A inhibition.

### Effects of LDH-A inhibition on the transcriptional levels of metabolic enzymes and hypoxia-inducible factors

**Figure 8:**
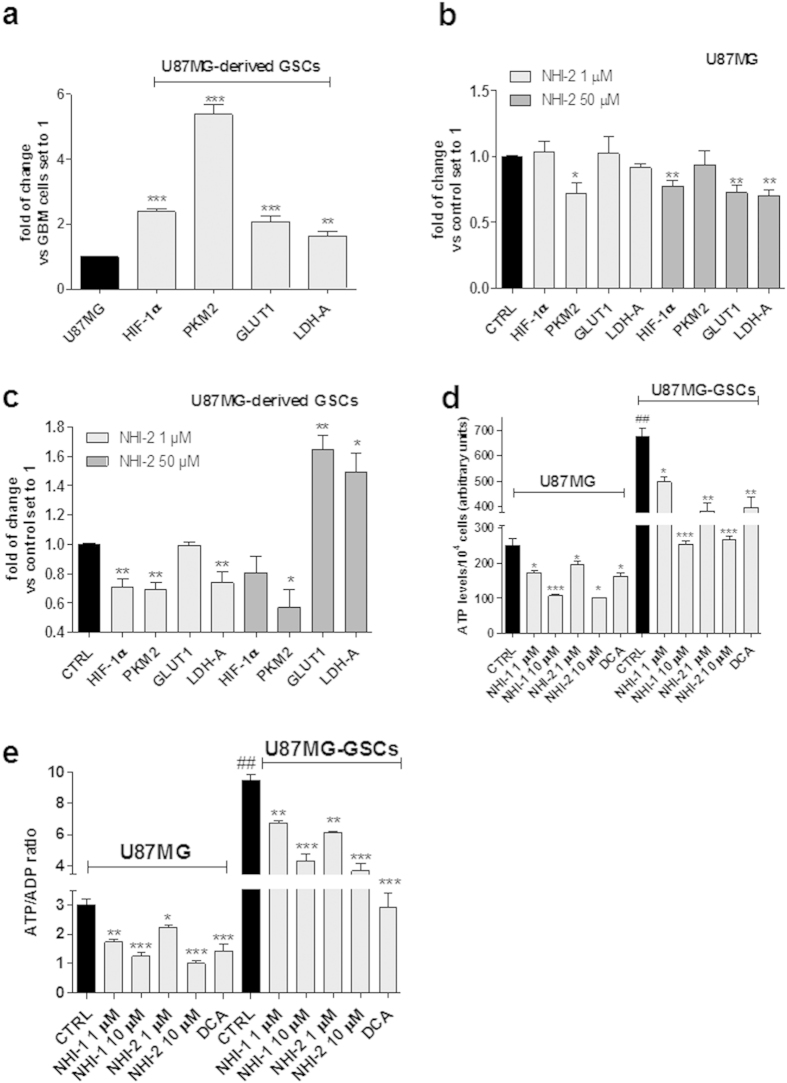
Effect of LDH-A inhibition on the expression of metabolic genes and on ATP/ADP ratio. (**a**–**c**) U87MG or U87MG-GSCs were treated with DMSO (control), or NHI-1 or NHI-2 (1 μM−50 μM) for 48 h or 7 days, respectively. At the end of the treatment periods, the mRNA amount of HIF-1α, PKM2, GLUT1 and LDH-A was quantified using real time RT-PCR. The data were expressed as the fold change relative to the level of expression in GBM cells or control set to 1, and are the mean values ± SEM of three different experiments performed in duplicate. (**d**,**e**) U87MG cells or U87MG-GSCs were treated with DMSO (control), or NHI-1 or NHI-2 (1 μM−10 μM), or DCA (100 μM) for 48 h or 7 days. At the end of treatments, ATP and ADP levels were quantified by a luminescence assay kit. The data were expressed as ATP levels (**d**) or ATP/ADP ratio (**e**), and are the mean values ± SEM of three different experiments performed in triplicate. The significance of the differences is determined with a one-way ANOVA with Bonferroni’s post hoc test: *P ≤ 0.05, **P ≤ 0.01, ***P ≤ 0.001 vs. respective control; ##P ≤ 0.01 vs. the relative expression in U87MG cells.

The transcriptional levels of metabolic enzymes, which play crucial role in the regulation of cancer cells metabolism, were examined in U87MG cells and in their derived GSCs. In particular, the attention was focused on LDH-A, on glucose transporter GLUT1 and on the M2 splice isoform of pyruvate kinase (PKM2), a low activity isoform of pyruvate kinase that promotes the aerobic glycolysis and contributes to anabolic metabolism^40^,^41^. The mRNA expression of LDH-A, GLUT1 and PKM2 was significantly higher in GSCs respect to that in U87MG cells (Fig. 8a). Similar results were obtained in U343MG, ANGM-CSS and T98G cells (Supplementary Figure 8).

Challenging U87MG cells with NHI-2 slightly, but significantly, reduced LDH-A mRNA expression only at 50 μM compound concentration (Fig. 8b). On the contrary, in GSCs, the effects of compound NHI-2 on LDH-A transcription appeared to be evident at the 1 μM concentration and are more marked with respect to GBM cells (Fig. 8c). Surprisingly, when NHI-2 was used at 50 μM, a significant increase in LDH-A mRNA expression was observed in the GSCs (Fig. 8c).

Interestingly, the expression of GLUT1 and PKM2 were differentially regulated by high concentrations of the drugs in the two cell populations. Indeed, treatment with NHI-2 caused a significant decrease of the PKM2 mRNA levels and an increase of GLUT1 and LDH-A mRNA levels in the GSCs (Fig. 8c), whereas a decrease of the GLUT1 and LDH-A expression was observed in U87MG cells (Fig. 8b).

It is well known that cells using aerobic glycolysis exhibit high ratios of ATP/ADP and NADH/NAD^+^ 41,42. Because the cytosolic ATP/ADP ratio is a key feature that determines if cell metabolism is predominantly oxidative or glycolytic^43^, the levels of ATP and ADP were measured in U87MG and in GSCs upon treatment with LDH-A inhibitors.

As depicted in Fig. 8d,e, ATP levels and ATP/ADP were significantly higher in GSCs with respect to U87MG cells, consistent with the greater glycolytic rate reported for GSCs^44^. Both NHI-1 and NHI-2 significantly reduced ATP content in U87MG cells (Fig. 8d), and decreased the ATP/ADP ratio (Fig. 8e), with an effectiveness comparable to DCA. These effects were even more evident in the GSCs (Fig. 8d,e), suggesting that ATP depletion may have a role in the GSC apoptosis elicited by LDH-A inhibition.

Several studies have demonstrated that GSCs are maintained within hypoxic niches and that hypoxia-inducible factors (HIFs), including HIF-1α and HIF-2α, have potential biological effects in maintaining the stemness of these cells^45^,^46^. Therefore, we investigated the modulation of the HIF-1α transcript levels upon incubation with NHI-2. The GSCs exhibited increased expression of HIF-1α compared to the differentiated U87MG cells (Fig. 8a), consistent with previously reported data^9^,^36^. Interestingly, the compound caused a significant reduction in the HIF-1α level after 7 days of treatment (Fig. 8c), consistent with the marked inhibition of GSC proliferation at this time point. In parallel, the compound was able to decrease significantly the gene expression of hypoxic inducible factor also in the normal U87MG cells after 48 h of treatment (Fig. 8b).

## Discussion

In this study, the inhibition of LDH-A activity proved to be an efficient strategy to target and control the GBM-derived cancer stem cells. LDH-A inhibition was demonstrated to reduce GSC viability and to elicit long-lasting effects, by triggering both apoptosis and cell differentiation. These data suggested that LDH-A is a promising pharmacological target for anti-CSC therapies, particularly for those intended to treat recurrent GBM.

GBMs up-regulate glucose uptake and aerobic glycolysis more than three times that of the normal brain tissue^47^. This metabolic divergence between GBM and normal cells may provide clues about novel therapeutic strategies that can be exploited. Any of steps in the glucose metabolism pathway may act as a putative therapeutic target, with various side effects. In this respect, although several papers have explored the benefits of targeting cancer metabolism in GBM^24^, the effects of direct LDH-A inhibition in this tumour have not yet been investigated, particularly in the stem cell population. LDH-A is required not only for tumour initiation but also for tumour maintenance and progression^22^,^23^; its inhibition was reported to block the progression of human lymphoma and pancreatic cancer xenografts^22^. Moreover, reduced LDH-A expression was recently demonstrated to impact the proliferation and self-renewal of adenocarcinoma stem cells^25^, thus supporting a mechanistic rationale by which inhibition of LDH-A could also be a viable therapeutic target for GSCs.

We found that the LDH-A inhibitors NHI-1 and NHI-2 altered the cell cycle progression and triggered apoptosis of different GBM cell lines, thus decreasing GBM proliferation. The inhibition of LDH activity was associated with a strong collapse of the mitochondrial membrane potential, consistent with the previously reported data in other tumours^23^. However, the inhibition of cell proliferation elicited by NHI-1 and NHI-2 was not long-lasting; in fact, U87MG cells partially recovered their growth after 72 h of wash-out, suggesting that these compounds are not able to permanently inhibit the growth/survival of GBM cells.

Then, the effects of LDH-A inhibition in GSCs isolated from U87MG cells were examined. Indeed, recurrence of GBM growth is attributed to the presence of treatment-resistant GSCs: targeting these cells may therefore be an effective strategy to improve GBM treatment outcome. GSCs are highly dependent on glycolysis, more so than their respective differentiated tumour cells, and they exhibit low mitochondrial respiratory activity^9^, potentially rendering them more sensitive to therapeutic strategies based on the inhibition of the glycolytic pathway^10^. Furthermore, LDH-A is overexpressed in breast CSCs, which exhibit an increased LDH (Pyr-to-Lac) activity and lactic acid levels, thus supporting the aforementioned hypothesis.

First, we showed that both LDH-A inhibitors induced a time-dependent inhibition of GSC proliferation, induced a cell cycle block and triggered their apoptosis. NHI-2 induced a concentration-dependent inhibition of GSC proliferation, yielding a higher potency in GSCs with respect to U87MG cells. Similar results were obtained in different GBM cell lines (i.e., U343MG and ANGM-CSS). Interestingly, lower effects were obtained in the p53 mutated-T98G cells, thus suggesting that the p53 pathway may have a considerable role in LDH-A inhibition-mediated effects, consistent with data previously reported^30^.

Of note, wash-out experiments evidenced the overall inability of GSCs to recover their normal growth after challenging with the two compounds, suggest the ability of LDH-A inhibitors to preferentially produce long-lasting effects in the GSC subpopulation.

To investigate the mechanism underlying these effects, the morphology and stemness/differentiation degree of GSCs upon treatment with the compounds were evaluated. Both NHI-1 and NHI-2 caused a reduction in the area occupied by GSC spheres, and the prominent outgrowth of cellular processes. Real-time RT-PCR and western blotting analyses confirmed that the two compounds significantly promoted GSC differentiation toward a neuronal and a glial phenotype. These results demonstrate that a LDH-A inhibition is able to reduce the stemness of GSC, and to promote a differentiated phenotype, which most likely presents a lower glycolytic rate.

Then, the transcriptional levels of metabolic enzymes, which play a crucial role in the regulation of cancer cells metabolism, were examined in U87MG cells and in their derived GSCs. The expression of LDH-A, GLUT1 and PKM2 was significantly higher in GSCs respect to U87MG cells, consistent with the metabolic switch to glycolysis occurring during the transformation into CSCs^9^,^36^.

Challenging GBM with 50 μM NHI-2 slightly, but significantly, reduced the LDH-A mRNA expression. In GSCs the compound, tested at 1 μM, decreased LDH-A transcription as well, with a higher rate of reduction than that observed in the GBM cells, consistent with the data reported in adenocarcinoma CSCs^25^.

Treatment with NHI-2 significantly decreased PKM2 mRNA levels, which is consistent with the pro-differentiation effects promoted by this compound. Indeed, PKM2 is a low-activity isoform of pyruvate kinase that promotes aerobic glycolysis^40^,^41^ and is down-regulated in cancer cells compared to their CSC subpopulation^9^. Conversely, both GLUT1 and LDH-A transcript levels were increased in GSCs upon challenge with a high concentration of NHI-2. This paradoxical effect could be attributed to a compensatory mechanism of GSCs after glycolysis depletion, by which the cells try to escape from the inhibition of the glycolytic pathway by increasing glucose uptake.

Surprisingly, expression of the two metabolic enzymes was differently regulated in the U87MG cells upon LDH-A inhibition: whereas there were no significant effects on PKM2 mRNA levels, a decrease in GLUT1 mRNA production was observed. These differences may account for the higher susceptibility of GSCs to LDH-A inhibition.

GSCs exhibited higher ATP levels and ATP/ADP ratio with respect to U87MG cells, consistent with the higher glycolytic rate previously reported for GSCs^44^.

Herein, we demonstrated that LDH-A inhibitors reduced both ATP content and ATP/ADP ratio in U87MG and in GSCs, thus suggesting that ATP depletion may have a significant role in GSC apoptosis elicited by LDH-A inhibition. Consistent with this hypothesis, 2-deoxyglucose (2-DG), a glucose analogue that is phosphorylated to 2-DG-phosphate, was reported to inhibit glycolysis, deplete ATP and promote cell cycle inhibition and cell death^48^,^49^. Moreover, the combination of bromo-2-oxopropionate-1-propyl ester with carmustine was shown to effectively kill GSCs through rapid depletion of cellular ATP levels^44^.

Finally, the effect of LDH-A inhibition on the expression of HIF-1α was assessed. Both NHI-1 and NHI-2 decreased the level of the HIF-1α transcript at 7 days of treatment. This result is consistent with the data previously reported in the literature, showing that silencing the HIF-1α gene resulted in the inhibition of GBM tumour growth, by both inhibiting the rate of tumour-cell migration/invasion and inducing CSC differentiation^50^,^51^. In line with these data, lactate and pyruvate were found to upregulate hypoxia inducible genes independently of hypoxia by stimulating the accumulation of HIF-1α in breast CSCs^52^. HIF-1α triggers the up-regulation of genes that are critical for the promotion of glycolysis, including GLUT1 and LDH-A, thus favouring the metabolic switch toward the non-oxidative pathway and, consequently, making the cells more sensitive to classical chemotherapeutics.

As reported by Xie and collaborators, the CSCs could be more vulnerable to LDH-A blockade due to the overall lower capacity of their Krebs cycle, despite a low glycolytic capacity, relative to the non-CSCs^25^. Accordingly, we demonstrated GBM-derived CSC are sensitive to glycolytic inhibition, and shed light on the therapeutic potential of LDH-A inhibitors in this types of tumour, which is characterised by a poor prognosis. Additional studies are in progress to further investigate the metabolic switch in GSCs upon treatment with LDH-A inhibitors in both normoxic and hypoxic conditions, as well as the putative use of these compounds in combination with conventional GBM chemotherapeutics.

## Materials and Methods

### GBM cell-lines and GSC isolation

The U87MG, T98G and U343MG cell lines were obtained from the National Institute for Cancer Research of Genoa (Italy), American Type Culture Collection (USA) and Cell Lines Service GmbH (Germany), respectively. ANGM-CSS cells were kindly gifted by IRCCS Casa Sollievo della Sofferenza Hospital, Italy. Each cell line was monitored for DNA profiling and cultured as described^34^,^53^.

To isolate GSCs from each GBM cell line, approximately 2.5 × 10^6^ U87MG cells were suspended in 1 mL of a defined serum-free Neural Stem Cell (NSC) medium^37^. After 3–5 days of culture, the GSCs (called “neurospheres”) were collected, suspended in NSC medium, dissociated into single cells and plated for the assays. The method accuracy for GSC isolation was verified in previous experiments^37^ and further confirmed by Real Time RT-PCR and western blotting analyses of stem cell (CD133, Nestin) and glial (GFAP) markers (see Supplementary Information). For the long-term treatment of cells, NSC or complete medium containing drugs was replaced every two to three days.

### Cell viability assays of GBM cells and GSCs

The human U87MG cells or GSCs were seeded at a density of 2.5 × 10^3^ cells/well. After 24 h, the cells were treated for 1 to 7 days with fresh growth medium containing different concentrations of the LDH-A inhibitors NHI-1 and NHI-2, synthesized as previously described^26^. Where indicated, DCA (100 μM) was used as a positive control. Following the treatment period, cell viability was determined using the Muse™ Count & Viability Reagent (Merck KGaA, Darmstadt, Germany) according to manufacturer’s instruction. For wash-out experiments, U87MG cells or GSCs were treated with NHI-1 and NHI-2 (1−10 μM) for 72 h (U87MG cells) or 7 days (GSCs). At the end of treatments, medium-containing drugs was replaced by fresh medium, and cells were allowed to growth for the indicated days (3 days in the case of U87MG, 7 days in the case of GSCs). At the end of treatments, cell proliferation was measured as described above. Data were analysed using the Muse^™^ Cell Analyzer software, and the results expressed as the percentage of untreated cells. Sigmoid dose-response curve was generated, from which the IC_50_ values were derived.

### Mitochondrial membrane potential (Δψm) dissipation analysis

The Δψm dissipation was assessed using the fluorescent dye 5,59,6,69-tetrachloro1,19,3,39-tetraethylbenzimidazolylcarbocyanine iodide (JC-1)^54^. U87MG or GSCs were treated with DMSO (control), or a high unique concentration (50 μM) of NHI-1 or NHI-2 for 24 h or 7 days, respectively. At the end of treatments, mitochondria were isolated from U87MG cells and GSCs using the Mitochondria Isolation Kit (Sigma Aldrich, Milan, Italy) following manufacturer’s instructions, and the JC-1 staining procedure was performed as follows. In a 96 well plate with black bottom, 90 μl of the JWS and 10 μl (5 μg of proteins) of isolated mitochondria were added. The fluorescence (relative fluorescence units, RFU) of the samples was read in a spectrofluorimeter using a time-drive method (one acquisition each 10 sec, for 10 minutes), with settings excitation wavelength at 490 nm and emission wavelength at 590 nm.

### RNA extraction and Real Time PCR analysis in U87MG cells and in GSCs

U87MG cells or GSCs were treated with DMSO (control), 10 μM NHI-1 or 10 μM NHI-2 for 48 h or 7 days, respectively. At the end of treatments, cells were collected, and total RNA was extracted using Rneasy® Mini Kit (Qiagen, Hilden, Germany) according to manufacturer’s instructions. cDNA synthesis was performed with 500 ng of RNA using i-Script cDNA synthesis kit (BioRad, Hercules, USA) following manufacturer’s instructions. RT-PCR reactions consisted of 25 μL Fluocycle® II SYBR® (Euroclone, Milan, Italy), 1.5 μL of both 10 μM forward and reverse primers, 3 μL cDNA, and 19 μL of H_2_O. All reactions were performed for 40 cycles using the following temperature profiles: 98 °C for 30 seconds (initial denaturation); T °C (see Suppl. Table 1) for 30 seconds (annealing); and 72 °C for 3 seconds (extension)^37^.

### Cell cycle analysis

U87MG or GSCs were treated with DMSO, 10 μM NHI-1 or NHI-2 for 48 h or 7 days, respectively. The measurement of the percentage of cells in the different cell phases was performed using the Muse™ Cell Analyzer (Merck KGaA, Darmstadt, Germany) as described previously^54^.

### Annexin V and 7-AAD staining in U87MG cells and in GSCs

U87MG cells or GSCs were treated with DMSO (control), NHI-1 or NHI-2 (1 μM−10 μM) for 48 h or 7 days, respectively. At the end of the treatment periods, the percentages of living, apoptotic and dead cells were acquired and analyzed by Muse™ Cell Analyzer as described previously^54^.

### Neurosphere formation assay

The ability of cells of monolayer cultures to initiate neurosphere formation were assessed by harvesting, washing, and resuspending monolayer cells in serum-free NSC medium. Cells were seeded into 96-well at 2 × 10^4^ cells/well and incubated with DMSO (0.5%, control) or compounds NHI-1 and NHI-2 (100 nM−1 μM) for 9 days without disturbing the plates and without replenishing the medium. At the end picture of the neurospheres were taken. Three different wells were analysed for each condition and 3 images of each well were captured. The number and the diameter of the newly formed neurosphere were counted using the Image J program (version 1.41; Bethesda, MD, USA).

### Quantitation of the occupied area and the cellular processes of neurospheres

GSCs were plated in complete growth medium (day 0) and treated for the indicated days with a concentration range from 10 nM to 50 μM of both compounds. At the end of the treatment periods, the drug-containing media were replaced with fresh NSC medium, and the GSCs were allowed to grow for another 7 or 14 days. Photographs of the neurospheres were taken at days 0, 7, and 21. Three different wells were analysed for each condition, and 15 images of each well were captured^37^,^55^. The response of the cultures to the various treatments was quantified by measuring the area occupied by neurospheres that had formed, using the Image J program (version 1.41; Bethesda, MD, USA). The cellular processes extending from the 6 to 8 differentiating neurospheres per condition in three independent experiments were evaluated.

### Western blotting analysis

U87MG cells or GSCs were treated with DMSO (control), NHI-1 or NHI-2 (10 μM) for 48 h or 7 days, respectively. At the end of the treatment periods, the cells were collected and lysed. Equal amounts of the cell extracts (40 μg of protein) were diluted in Laemmli sample solution, resolved using SDS-PAGE, transferred to PVDF membranes and probed overnight at 4 °C using the following primary antibodies: anti-nestin (sc-20978, Santa Cruz Biotechnology, Heidelberg, Germany; 1:150); anti-GFAP (sc-9065, Santa Cruz Biotechnology; 1:50); anti-NeuN (ab177487, Abcam, Cambridge, UK); and β-actin (MAB1501, Merck KGaA, Darmstadt, Germany)). The primary antibodies were detected using the appropriate peroxidase-conjugated secondary antibodies, which were then detected using a chemioluminescent substrate (ECL, Perkin Elmer). Densitometric analysis of the immunoreactive bands was performed using Image J Software.

### Measurement of ATP/ADP ratio

U87MG cells or GSCs were treated with DMSO (control), NHI-1 or NHI-2 (1 μM−10 μM) for 48 h or 7 days, respectively. At the end of the treatment periods, ATP and ADP levels were quantified by a luminescence assay kit (ApoSENSORTM ADP/ATP Ratio Assay Kit, Enzo Life Sciences, Vinci-Biochem, Florence, Italy).

### Statistical analysis

The nonlinear multipurpose curve-fitting program Graph-Pad Prism (GraphPad Software Inc., San Diego, CA) was used for data analysis and graphic presentations. All data are presented as the mean ± SEM. Statistical analysis was performed by one-way analysis of variance (ANOVA) with Bonferroni’s corrected t-test for post-hoc pair-wise comparisons. P < 0.05 was considered statistically significant.

## Additional Information

**How to cite this article**: Daniele, S. *et al.* Lactate dehydrogenase-A inhibition induces human glioblastoma multiforme stem cell differentiation and death. *Sci. Rep.* **5**, 15556; doi: 10.1038/srep15556 (2015).

## Supplementary Material

Supplementary Information
