# Supplementary material for: Lactate dehydrogenase-A inhibition induces human glioblastoma multiforme stem cell differentiation and death
Source: Sci Rep. 2015 Oct 23;5:15556. doi: 10.1038/srep15556 (PMC4616042; doi:10.1038/srep15556)
Supplement: Supplementary Information [file srep15556-s1.doc]

**Supplementary Information**

**Lactate dehydrogenase-A inhibition induces human glioblastoma multiforme stem cell differentiation and death**

Simona Daniele^1†^, Chiara Giacomelli^1†^, Elisa Zappelli^1^, Carlotta Granchi^1^, Maria Letizia Trincavelli^1*^, Filippo Minutolo^1^, Claudia Martini^1^.

**
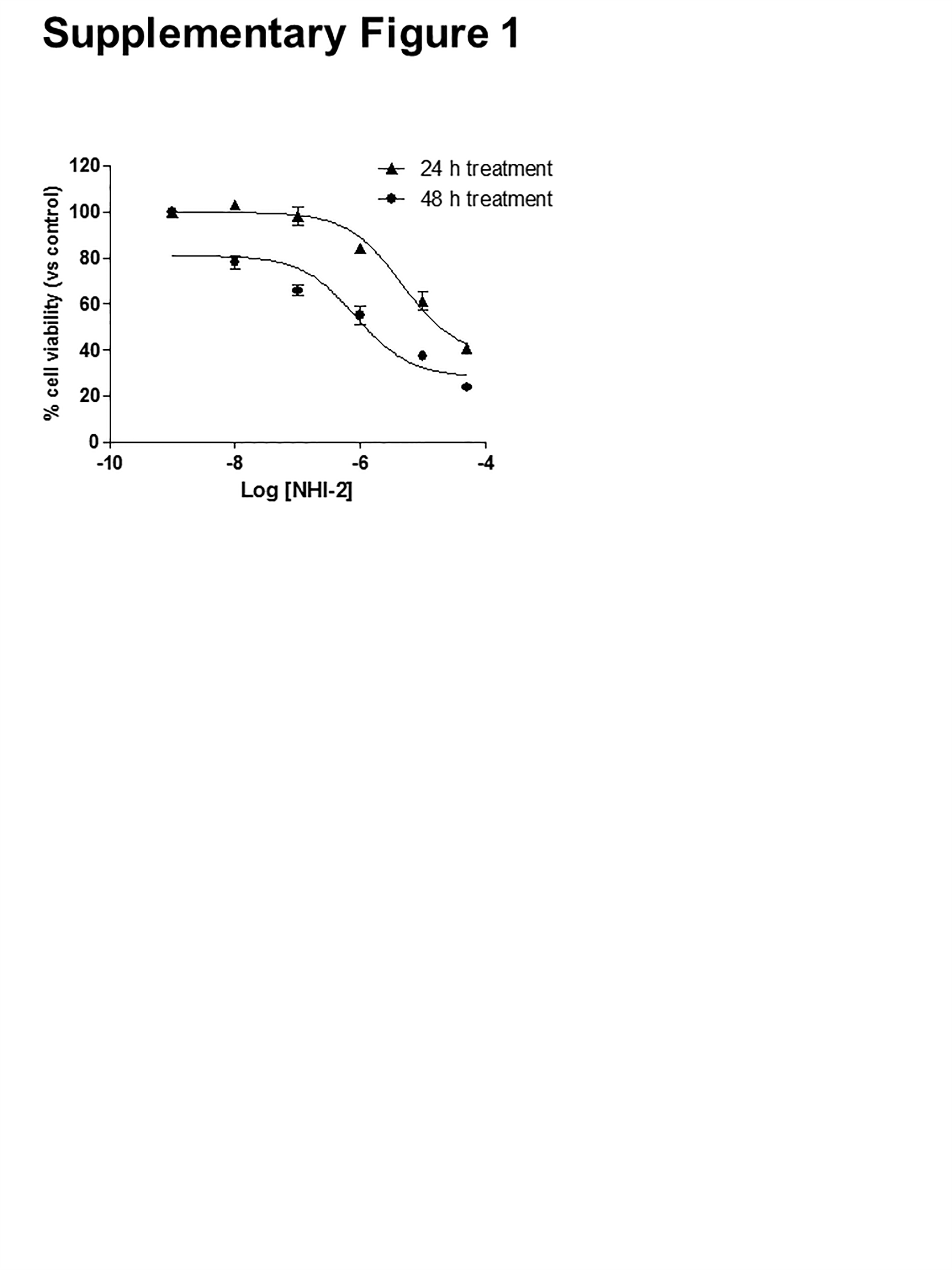
**


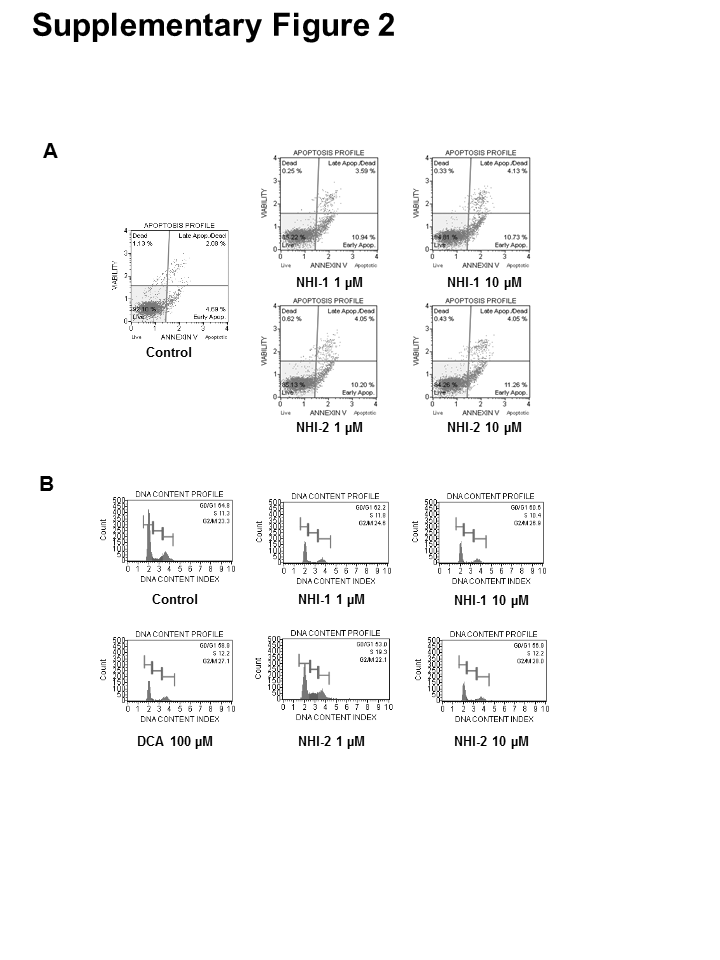


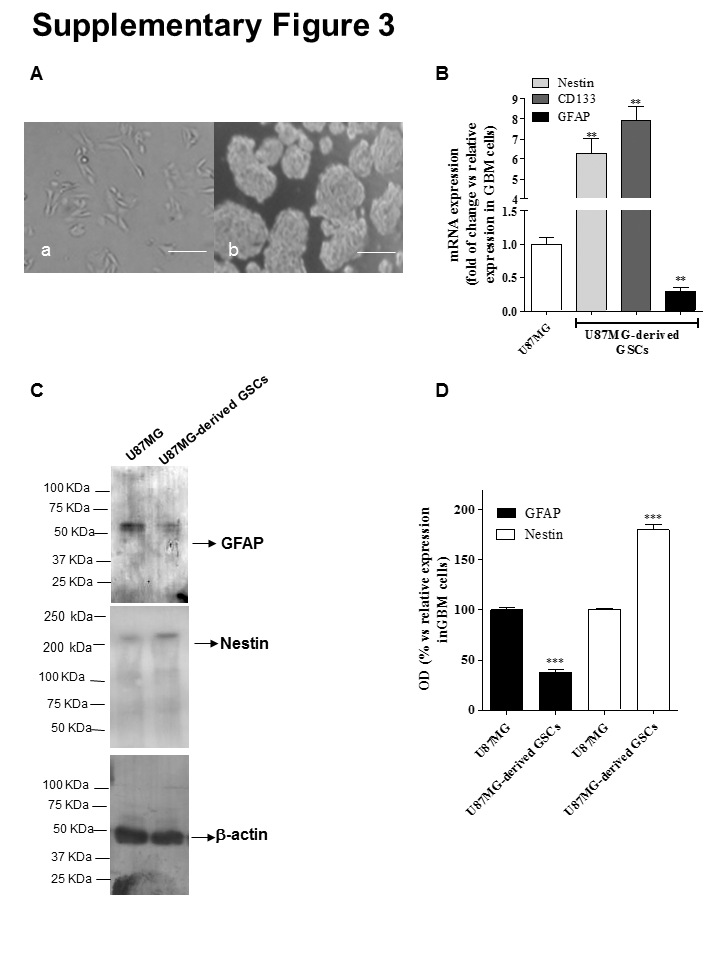


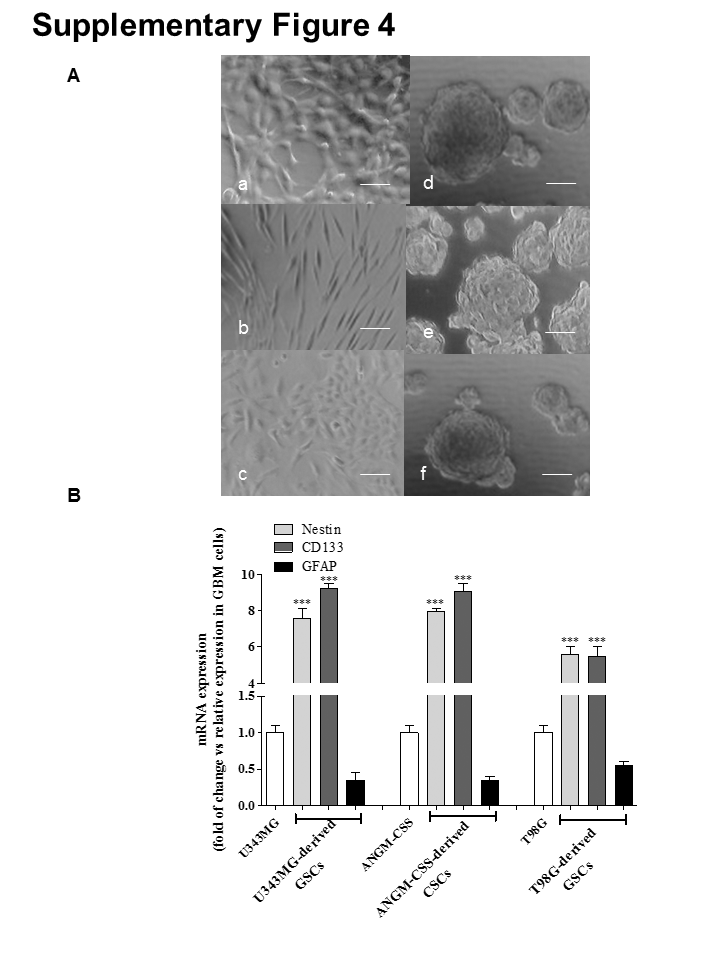


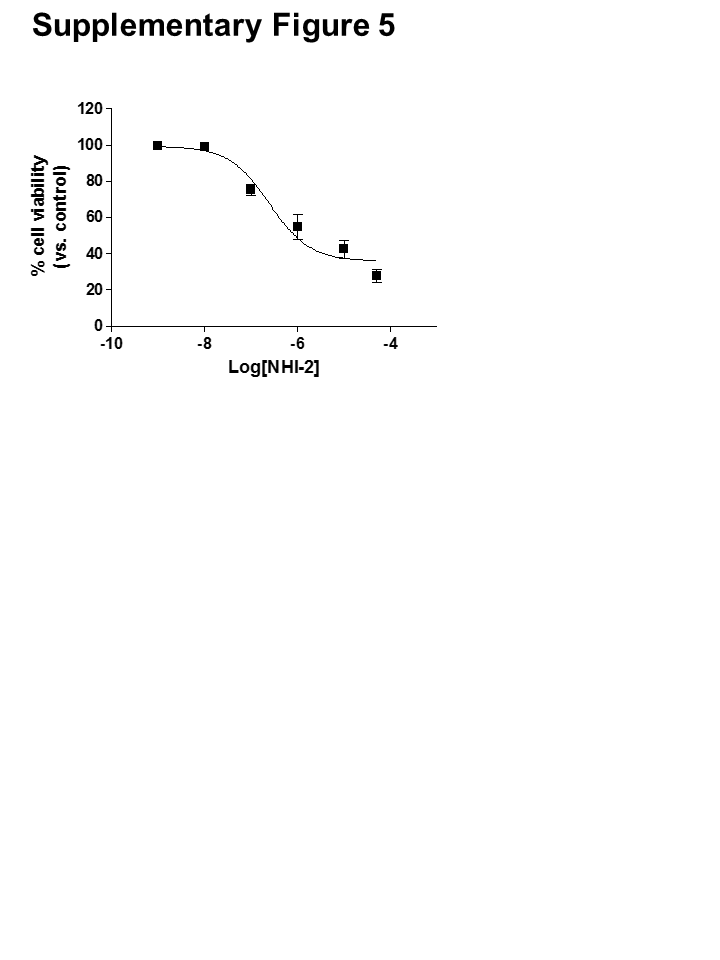


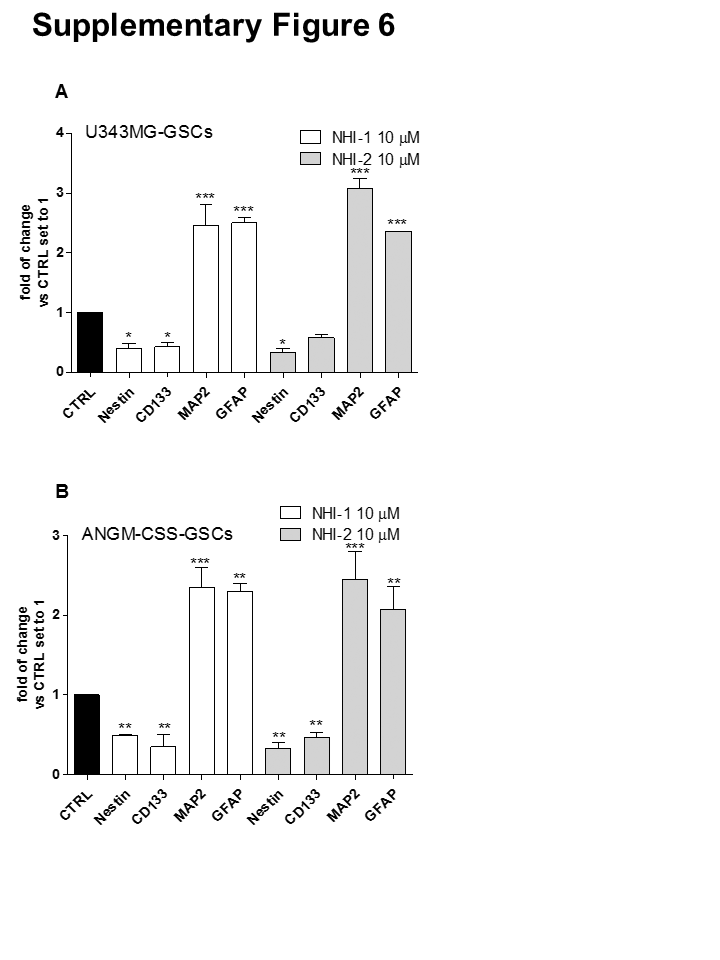

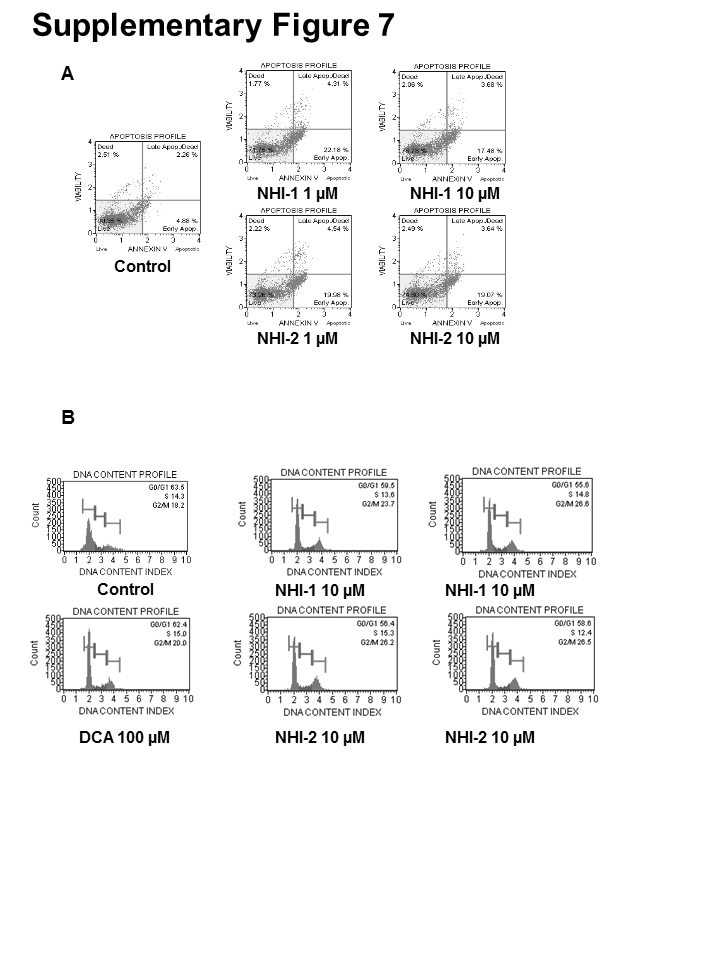

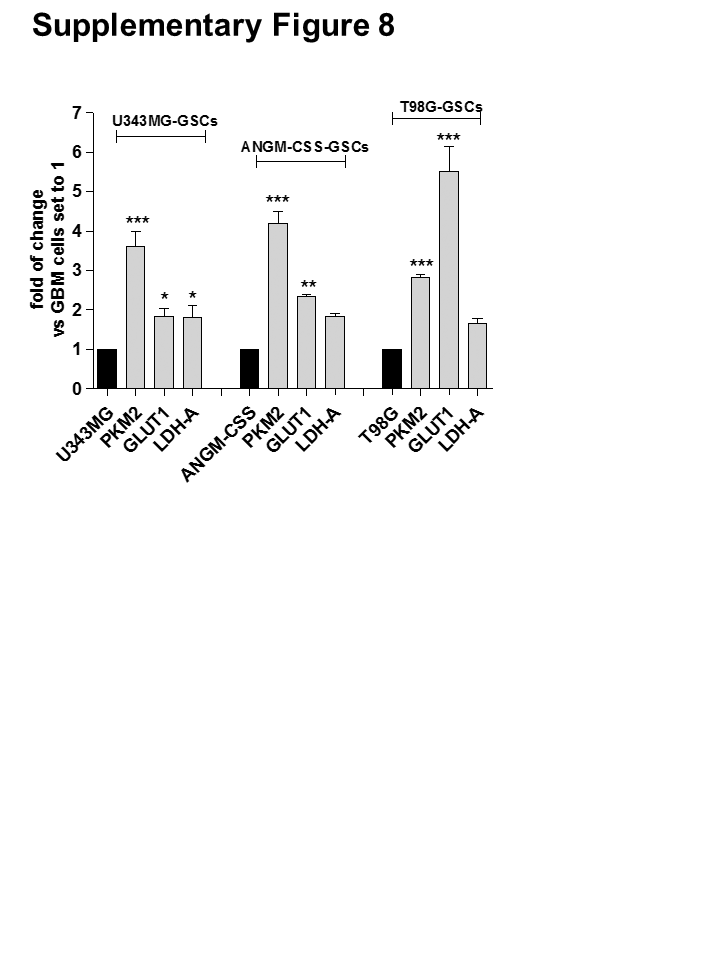


**‘‘Full-length blots relative to the cropped images showed in the main Figures’’**

**Figure 4**


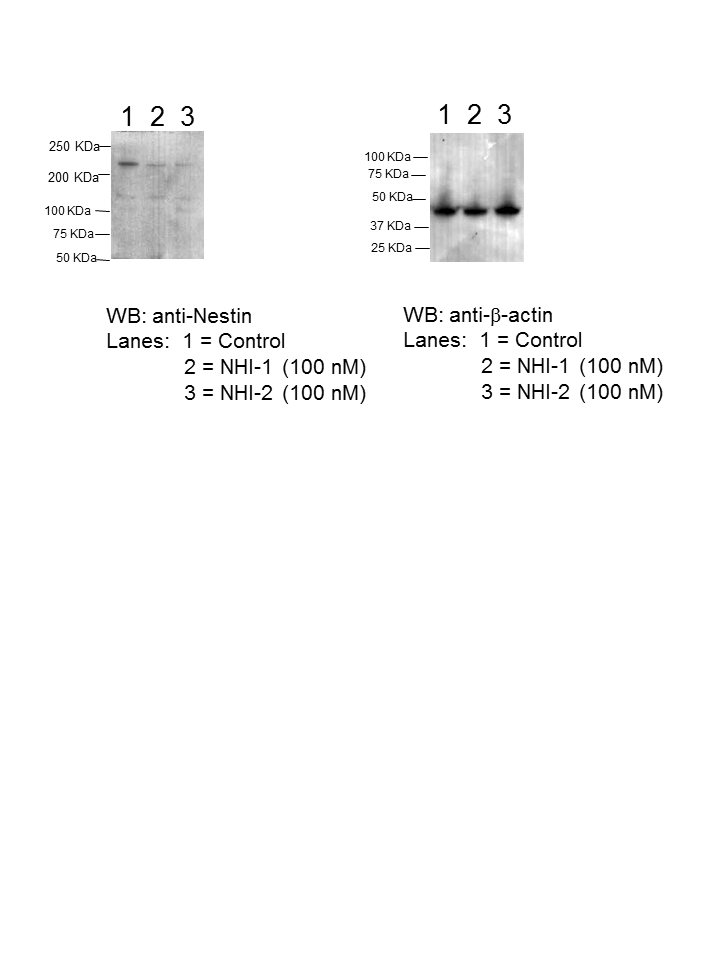


**‘‘Full-length blots relative to the cropped images showed in the main Figures’’**

**Figure 6**

**
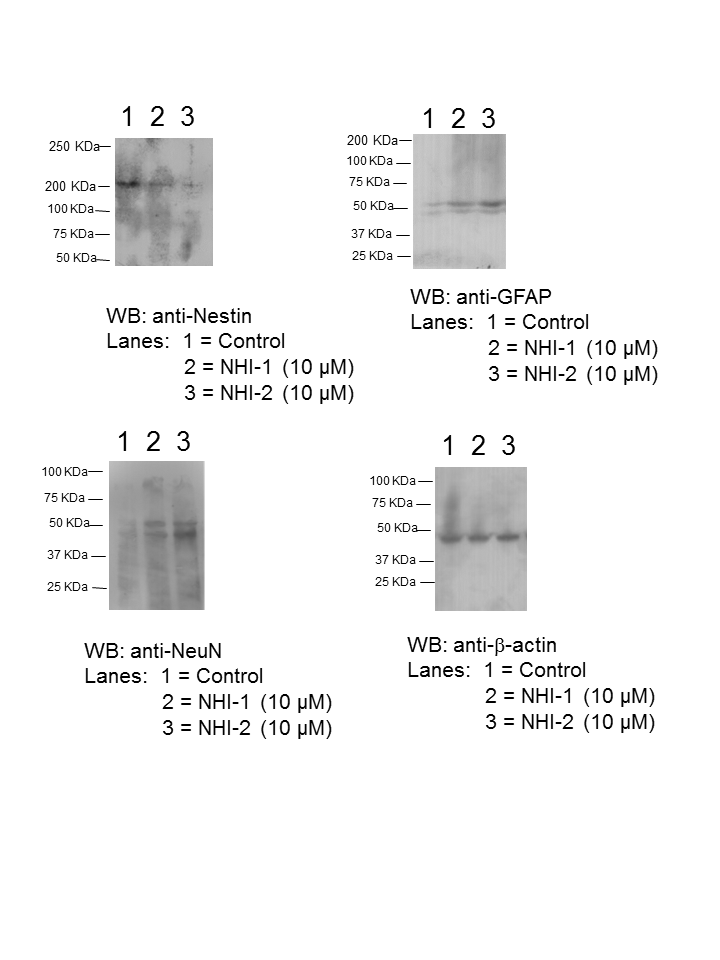
**

| **Gene** | **Primer nucleotide sequences** | **Product size**  **(base pairs)** | **Annealing Temperature** |
| --- | --- | --- | --- |
| CD133 | FOR: 5’-TCCACAGAAATTTACCTACATTGG -3’  REV: 5’-CAGCAGTTCAAGACGCAGATGACCA-3’ | 251 | 55°C |
| MAP2 | FOR: 5’-TTGGTGCCGAGTGAGAAGAA -3’  REV: 5’-GGTCATGCTGGCAGTGGTTGGT -3’ | 280 | 55°C |
| NESTIN | FOR: 5’-CAGCGTTGGAACAGAGGTTGG -3’  REV: 5’-TGGCACAGGTGTCTCAAGGG -3’ | 282 | 61°C |
| GFAP | FOR: 5’-CCTCTCCCTGGCTCGAATG -3’  REV: 5’-GACAACCGCCACTCAACTAGC-3’ | 287 | 52°C |
| PKM2 | FOR: 5’- GTACCATGCGGAGACCATCA-3’  REV: 5’- GTAGGCGTTATCCAGCGTGA-3’ | 199 | 55°C |
| GLUT1 | FOR: 5’- GCGGAATTCAATGCTGATGAT-3’  REV: 5’- CAGTTTCGAGAAGCCCATGAG-3’ | 70 | 55°C |
| HIF-1α | FOR: 5’-AAAGGACAAGTCACCACAGG -3’  REV: 5’-TTCTGTTTGTTGAAGGGAG -3’ | 216 | 50°C |
| LDH-A | FOR: 5’-AGCCCGATTCCGTTACCT-3’  REV: 5’-CACCAGCAACATTCATTCCA-3’ | 124 | 55°C |
|    β-actin | FOR: 5’-GCACTCTTCCAGCCTTCCTTCC-3’  REV-5’-GAGCCGCCGATCCACACG-3’ | 254 | 55°C |

**Table 1. Nucleotide sequences, annealing temperature and product size of the primers utilized in Real Time PCR experiments.**

**Supplementary figure legends**

**Supplementary Figure 1.** Effects of LDH-A inhibition on U87MG cell viability. U87MG were treated for 72 h with the indicated concentrations of **NHI-2**. At the end of the treatment, cell viability was measured using a CellTrace dye labelling, as described in the Methods section, and non-linear regression of **NHI-2** dose-response curve was calculated. Data were expressed as percentage with respect to that of untreated cells (control), which was set to 100%, and they are the mean values ± SEM of three independent experiments, each performed in duplicate.

**Supplementary Figure 2.** Effects of LDH-A inhibition on U87MG apoptosis and cell cycle. (**A**) U87MG cells were treated for 48 h with DMSO (control), or **NHI-1** or **NHI-2**, at 1 µM or 10 µM. At the end of the treatments, the cells were collected and the level of phosphatidylserine externalisation was evaluated using the Annexin V-staining protocol, as described in the Methods section. Representative histograms of untreated and treated cells were shown. (**B**) U87MG cells were treated as in A for 72h. At the end of the treatment, the cells were collected and the cell cycle was analyzed as described in the Methods section. Representative cell cycle histograms of untreated and treated cells were shown.

**Supplementary Figure 3.** Characterization of GSCs derived from U87MG cells. **A)** Representative bright field microscope images showing whole U87MG (*a*), and the derived GSCs (*b*). **B)** The total RNA was extracted from U87MG cells and from the derived GSCs. The relative mRNA quantification of the stem cell markers (CD133 and Nestin) and of the astrocyte marker GFAP was performed by Real-time PCR, as described in the Methods section. The data were expressed as the fold change relative to the level of expression in U87MG cells, and they are the mean values ± SEM of two different experiments. **C, D)** Cell lysates were prepared from U87MG cells and the derived GSCs, and then GFAP and nestin protein levels were evaluated by Western blot analysis. GAPDH was the loading control. C) Representative Western blots. D) Densitometric analysis of the immunoreactive bands performed using ImageJ program. The data were expressed as the percentage relative to the level of expression in whole GBM cells, and they are the mean values ± SEM of three different experiments. Statistical significance was determined with a one-way ANOVA with Bonferroni’s post hoc test: **P≤0.01, ***P≤0.001 vs the relative expression in GBM cells.

**Supplementary Figure 4.** Characterization of GSCs derived from U343MG, ANGM-CSS and T98G cells**. A)** Representative bright field microscope images showing whole U343MG (*a*), ANGM-CSS (*b*), T98G (*c*) cells and the respective derived GSCs (*d, e, f*). **B)** The total RNA was extracted from U87MG cells and from the derived GSCs. The relative mRNA quantification of the stem cell markers (CD133 and Nestin) and of the astrocyte marker GFAP was performed by Real-time PCR, as described in the Methods section. The data were expressed as the fold change relative to the level of expression in GBM cells, and they are the mean values ± SEM of three different experiments. Statistical significance was determined with a one-way ANOVA with Bonferroni’s post hoc test: ***P≤0.001 vs the relative expression in GBM cells.

**Supplementary Figure 5.** Effect of LDH-A inhibition on GSC viability. GSCs were incubated for 7 days with increasing concentrations of **NHI-2**, and cell viability was measured using a CellTrace dye labelling, as described in the Methods section. The dose-response curve of **NHI-2** on GSCs treated for 7 days is shown. Data were expressed as percentage with respect to that of untreated cells (control), which was set to 100%, and they are the mean values ± SEM of three independent experiments, each performed in duplicate.

**Supplementary Figure 6.** Effect of LDH-A inhibition on GSC differentiation. GSCs isolated from U343MG (**A**) or ANGM-CSS (**B**) were treated for 7 days with complete NSC medium containing DMSO (control), or **NH-1** or **NHI-2** (10 μM). At the end of treatments, total RNA was extracted; the relative mRNA quantification of the stem cell marker (CD133 and Nestin), the neuronal marker MAP2 and of the astrocyte marker GFAP were performed by real time RT-PCR. Data were expressed as fold of change vs the levels of the control set to 1 and are the mean values ± SEM of three different experiments. The significance of the differences was determined with a one-way ANOVA with Statistical significance was determined with a one-way ANOVA with Bonferroni’s post hoc test: *P ≤ 0.05, ** P ≤ 0.01, ***P ≤ 0.001 vs control.

**Supplementary Figure 7.** Effects of LDH-A inhibition on GSC apoptosis and cell cycle. (**A**) GSCs cells were treated for 7 days with DMSO (control), or **NHI-1** or **NHI-2**, at 1 µM or 10 µM. At the end of the treatments, the cells were collected and the level of phosphatidylserine externalisation was evaluated using the Annexin V-staining protocol, as described in the Methods section. Representative histograms of untreated and treated cells were shown. (**B**) GSCs were treated as in A. At the end of the treatment periods, the cells were collected and the cell cycle was analyzed, as described in the Methods section. Representative cell cycle histograms of untreated and treated cells were shown.

**Supplementary Figure 8.** Expression of metabolic genes in U343MG, ANGM-CSS, T98G and relevant GSCs. U343MG, ANGM-CSS, T98G and the respective GSCs were collected. The mRNA amount of, PKM2, GLUT1 and LDH-A was quantified using real time RT-PCR, as described in the Methods section. The data were expressed as the fold change relative to the level of expression in GBM cells set to 1, and are the mean values ± SEM of three different experiments performed in duplicate. The significance of the differences was determined with a one-way ANOVA with Statistical significance was determined with a one-way ANOVA with Bonferroni’s post hoc test: * P≤0.05, ** P≤0.01, *** P ≤0.001 vs GBM cells.
